# Inflammasome-Mediated Cytokines: A Key Connection between Obesity-Associated NASH and Liver Cancer Progression

**DOI:** 10.3390/biomedicines10102344

**Published:** 2022-09-21

**Authors:** Nathalia Soares da Cruz, Gabriel Pasquarelli-do-Nascimento, Augusto Cézar Polveiro e Oliveira, Kelly Grace Magalhães

**Affiliations:** Laboratory of Immunology and Inflammation, Department of Cell Biology, University of Brasilia, Brasilia 70910-900, Brazil

**Keywords:** HCC, NASH, obesity, inflammation, inflammasomes

## Abstract

Liver cancer is one of the most lethal malignancies and is commonly diagnosed as hepatocellular carcinoma (HCC), a tumor type that affects about 90% of patients. Non-alcoholic steatohepatitis (NASH) and obesity are both risk factors for this disease. HCC initiation and progression are deeply linked with changes in the hepatic microenvironment, with cytokines playing key roles. The understanding of the pathogenic pathways that connect these disorders to liver cancer remains poor. However, the inflammasome-mediated cytokines associated with both diseases are central actors in liver cancer progression. The release of the pro-inflammatory cytokines IL-1β and IL-18 during inflammasome activation leads to several detrimental effects on the liver microenvironment. Considering the critical crosstalk between obesity, NASH, and HCC, this review will present the connections of IL-1β and IL-18 from obesity-associated NASH with HCC and will discuss approaches to using these cytokines as therapeutic targets against HCC.

## 1. Introduction

According to data from the Global Burden of Disease (GBD), in 2019 liver cancer was the eighth death-related type of cancer in the world. Hepatocellular carcinoma (HCC) is the most common primary liver cancer and covers about 80–90% of cases [1]. This cancer is aggressive and impacts liver hepatocytes. Its diagnosis can be made through magnetic resonance imaging, ultrasound, and serological tests that identify biomarkers such as alpha-fetoprotein (AFP), des-gamma-carboxy-prothrombin (DCP) [2], and, more recently, micro RNAs, such as miR-25 [3]. Biopsy is another option, but it is avoided due to its invasiveness [2]. 

HCC impacts patients’ quality of life, and the most common symptoms are abdominal pain, weight loss, fever, and the worsening of hepatic synthetic function [4]. However, many patients are asymptomatic, and the diagnosis is made late when the disease is already advanced [5]. In addition, HCC, as with other cancers, is highly heterogeneous among patients due to genetic and epigenetic diversity and the tumor microenvironment [6], which makes treatment difficult. This heterogeneity is also reflected in the HCC immune response, which can be divided into some sub-classes, including “active immune”, “exhausted immune”, and “immune excluded” [7]. The “active immune” subtype associates with active T helper cells (CD4^+^) and CD8^+^ enrichment and responds well to treatment with immune checkpoint inhibitors (ICIs). The “exhausted immune” subtype is characterized by abundant TGFβ-secreting T lymphocytes, which are cells that show an exhausted status, and by the presence of immunosuppressive macrophages. In turn, the “immune excluded” subtype copes with increased an Treg cell number, has a worse prognosis, and does not respond to ICI therapies [1].

The main risk factors for HCC are infections by hepatitis B (HBV) and hepatitis C (HCV) viruses, alcohol abuse, obesity, and non-alcoholic steatohepatitis (NASH) [8]. NASH is part of a spectrum of liver disorders called non-alcoholic fatty liver diseases (NAFLD), which range from steatosis (NAFL), characterized by fat accumulation in liver tissue, to NASH, described as a pathological fat storage process associated with inflammation, liver injury, and fibrosis [9,10]. A “two-hit” model was proposed to explain NASH establishment [11]. The “first hit” is related to a dysregulated accumulation of lipids within liver tissue, generating hepatic steatosis, and the “second hit” triggers hepatocellular injury and inflammation due to liver oxidative stress and lipid peroxidation [11]. However, several studies have shown alternative mechanisms involving multiple pathways and metabolic hits that result in an unhealthy liver.

Within the NAFLD spectrum, only NASH, in association with fibrosis, can progress to cirrhosis and HCC [12,13]. Obesity is often present in patients with NASH, and this may increase the risk of developing HCC [14]. Additionally, studies have demonstrated that the gut microbiota plays crucial roles in nutrient harvest and fat storage, important processes for obesity progression [15], and deeply impacts NASH development, once patients show dysbiosis that favors alcohol-producing microbes and the occurrence of systemic inflammatory responses [16,17,18]. The connection between obesity-associated NASH pathways and HCC remains poorly understood. However, cumulative factors, such as inflammasome-mediated cytokines from obesity-associated NASH, are a possible link between these diseases with HCC development. 

Obesity is characterized by a low-grade chronic inflammation [19] with dysregulation in the adipose tissues (ATs), among them white AT (WAT) and brown AT (BAT), endocrine organs that can modulate the inflammatory status through the secretion of several mediators, such as chemokines and cytokines [20]. The latter includes the pro-inflammatory molecules interleukin-1β (IL-1β) and IL-18, which are maturated by the action of assembled inflammasomes, multiprotein complexes that can also induce immunogenic pyroptotic cell death [21]. The inflammasomes are crucial for inflammatory homeostasis, directly influencing both obesity and NASH as well as liver cancer [22]. Although inflammasomes were first related to the organism’s defense against pathogens, studies have linked these molecules to many metabolic diseases, such as obesity [22], type 2 diabetes mellitus [16], NAFLD [16,23], and HCC [24]. In this review article, we will present the possible links between inflammasome-mediated cytokines, obesity-associated NASH, and HCC development, and we will also discuss approaches to using IL-1β and IL-18 as therapeutic targets in the context of HCC.

## 2. Inflammasomes as a Mediator of Pro-Inflammatory Cytokines

Inflammation is a mechanism of the immune system to protect the host against pathogen-associated molecular patterns (PAMPs) and danger-associated molecular patterns (DAMPs). Innate immunity recognizes PAMPs and DAMPs by germline-encoded pattern-recognition receptors (PRRs). Ligand recognition or cellular disorder is a trigger to activate downstream signaling pathways followed by the production of pro-inflammatory cytokines and chemokines [25].

There are some classes of PRRs, among them the Toll-like receptor (TLR), a transmembrane protein situated on the cell surface, absent in melanoma 2 (AIM2), a sensor for cytosolic DNA [26], and NOD-like receptor (NLR), which recognizes PAMPs in the cytoplasm [27,28]. In humans, there are 22 members in the NLR family, categorized into 4 subfamilies based on N-terminal domains: NLRA, with a domain formed by acidic transactivation; NLRB, accompanied by a baculoviral inhibitory repeat (BIR) domain; NLRC, containing a caspase-recruitment and activation domain (CARD); and NLRP, a pyrin-containing domain (PYD). In addition to that, NLRs have an intermediary NACHT, NOD, or NBS domain and a C-terminal leucine-rich repeat (LRR) domain [29,30]. 

Currently, the NLR family members, including NLRP1, NLRP3, and NLRC4, are best described as assembled inflammasome components, as well as AIM2 and pyrin [30]. In 2002, Martinon and colleagues showed that the inflammasomes are responsible for activating the protease caspase-1 [31]. Once activated, this protein cleaves zymogen forms of IL-1β and IL-18 into their active form [32]. In addition to activating these potent pro-inflammatory cytokines, caspase-1 is also capable of inducing pyroptotic cell death [33]. The connection between NLRs and caspase-1 occurs through the adaptor protein ASC (apoptosis-associated speck-like protein, containing a CARD), which contains an N-terminal PYD domain and a C-terminal CARD domain [34]. Studies have demonstrated that the absence of ASC in mice alters the maturation and release of IL-1β and IL-18 [35,36,37]. Therefore, ASC has an important role in the inflammasome function [37]. 

The canonical pathway of NLRP3 inflammasome activation and the consequent release of IL-1β and IL-18 is a process tightly controlled by two signals. The first involves TLR stimulation, which results in the transcriptional upregulation of genes encoding the inflammasome components, pro-IL-1β and pro-IL-18, via nuclear factor kappa B (NFκB) activity. The second signal, needed for activating caspase-1, is provided by microparticles, such as ATP, via the P2X7 receptor, reactive oxygen species, K^+^ efflux through ion channels, and cathepsin B activation [38,39].

In turn, the non-canonical pathway occurs through direct binding of LPS with caspase-11 (in mice) and caspase-4/5 (in humans), without TLR activation [40]. Then, active caspase-11 will cleave the IL-1β and IL-18 cytokines. Caspase-11 is also involved in the pyroptotic cell death [41].

The inflammasome-mediated cytokines can lead to an inflammatory microenvironment in obesity that contributes to NASH establishment and may support liver cancer development.

## 3. IL-1β and IL-18 in Liver Cancer Progression

Inflammation has a dual effect in the cancer context, which can harm or benefit the tumor. Acute inflammation can trigger an anti-cancer immune response [42]. Chronic inflammation, on the other hand, can cope with augmented levels of cell growth and pro-angiogenic factors and with changes in the extracellular matrix that facilitate metastasis and DNA damage, thus contributing to an increase in cells with genetic alterations [43]. In the liver, IL-1β leads to, among other consequences, the release of IL-6 and TNF-α [44]. The increase in IL-6 levels is related to a worse prognosis in HCC patients [45]. In turn, IL-18 may influence the recruitment of T and NK cells [46].

There is a dysregulation in the NLRP3 inflammasome components in HCC depending on the stage of hepatocarcinogenesis [24]. Wei and others showed that during the development of HCC, the NLRP3 components are dysregulated depending on the disease stage, while in the inflammatory hepatic setting it copes with IL-1β and NLRP3 upregulation, and malignantly transformed liver cancer is downregulated [24]. IL-1β binds to interleukin-1 receptor type I (IL-1RI) and, after a series of cascades, activates NF-κB, which in turn is related to proliferation [47] and inflammation [48]. IL-1β can also promote the expression of the oncoprotein Gankyrin, which plays a critical role in HCC development and metastasis [49]. 

In 2019, Zong and colleagues demonstrated that M1 macrophages induced the expression of the programmed death ligand 1 (PD-L1) in HCC cells through IL-1β, supporting the pro-tumor role of M1 macrophages and IL-1β [50]. Corroborating with this study, another group recently showed that IL-1β induced PD-L1 expression in HCC, a phenomenon that contributes to tumor immune resistance in HCC [51]. Polymorphisms in the IL-1 family genes were described in HCC patients, which suggests that IL-1β contributes to HCC susceptibility and plays an important role in the progression of this neoplasm [52]. 

Another inflammasome-mediated cytokine, IL-18, is also involved in liver cancer occurrence. This cytokine is upregulated in HCV patients, an important risk factor for HCC. The studies showed that the IL-18 receptor (rhIL-18) is expressed in both HCC patients and cell lines [53]. The activation of this receptor was capable of increasing NF-κB activation and anti-apoptotic molecule expression, including Bcl-xL and xIAP [53]. Therefore, the expression of rhIL-18 and an antiapoptotic mechanism involving NF-κB activation in HCC cells may be related to poor prognosis in HCC patients [53]. Corroborating with these findings, a 2020 study indicated that IL-18 single nucleotide gene polymorphism could be a marker for HCC in patients with HCV-related cirrhosis [54], and IL-18 gene polymorphisms could be used as a potential non-invasive diagnostic tool for HCC patients at early stages [55], which is supported by recent findings that IL-18 high levels are found in HCC patients with poor prognosis [56].

**Figure 1:**
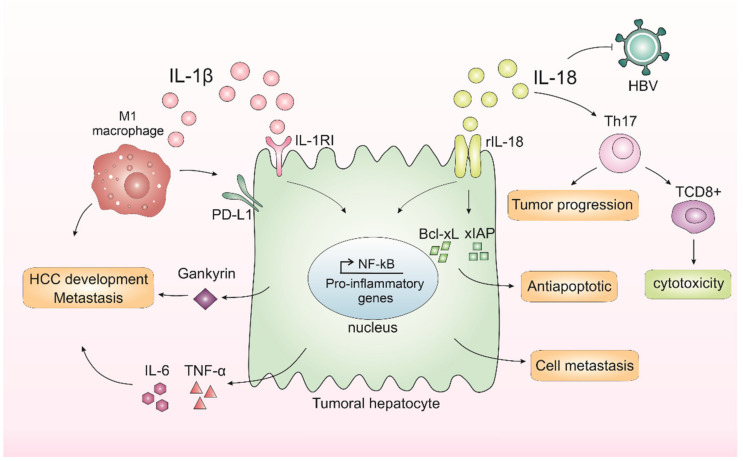
**Inflammasomes-mediated cytokines IL-1β and IL-18 in liver cancer progression.** In the liver, IL-1β leads to the release of IL-6 and TNF-α, cytokines related to a worse prognosis in hepatocellular carcinoma (HCC). IL-1β binds to interleukin-1 receptor type I (IL-1RI) and, after a series of cascades, activates nuclear factor kappa B (NF-κB), which in turn is related to proliferation and inflammation. IL-1β is also capable of promoting the expression of an oncoprotein, Gankyrin, which plays a critical role in HCC development and metastasis. M1 macrophages induce the expression of programmed death ligand 1 (PD-L1) in HCC cells through IL-1β, supporting the pro-tumor role of M1 macrophages and IL-1β. IL-18 receptor (rIL-18) is capable of increasing NF-κB activation and anti-apoptotic molecule expression, including Bcl-xL and xIAP. IL-18 can also regulate Th17 cells, which can both worsen prognosis or affect antitumor cytotoxicity by CD8^+^ T-cells. Finally, IL-18 could inhibit HBV replication but could also promote hepatocyte cell metastasis and migration.

In contrast, studies have also demonstrated a dual function of IL-18 in tumor progression. IL-18 can regulate Th17 cells in vitro and in vivo in the HCC model [57]. Th17 cells can both worsen prognosis [58,59] and affect antitumor cytotoxicity by CD8^+^ T-cells [59,60]. In human hepatocytes, it was demonstrated in vitro that IL-18 inhibited HBV replication but also promoted HepG2 cell metastasis and migration [59]. Taking these results, the IL-1β and IL-18 effects on HCC are demonstrated in Figure 1.

## 4. Obesity Chronic Inflammation as a Key in Liver Disorder

Obesity prevalence has been peaking at alarming rates [61], affecting more than 600 million adults worldwide [62]. The definition and classification of obesity are controversial; individuals presenting a body mass index (BMI) as 30 kg/m^2^ or higher are considered obese [63]. The occurrence of obesity epidemics is associated with an environment that promotes excessive food intake and insufficient levels of physical activity [64]. Social, economic, and behavioral aspects also contribute to the establishment and perpetuation of the obese phenotype [65]. As a consequence of the peaking rates related to the obesity-associated metabolic syndrome, liver disorders caused by this derangement show climbing statistics [66].

During obesity, liver fat accumulation occurs as a consequence of imbalanced fatty acid (FA) uptake and disposal. Insulin resistance (IR) in AT increases lipolysis and the release of these lipids, which are stored in the hepatic tissue [67]. This process is related to the establishment of NAFLD and its progressed inflammatory form called NASH, currently known as the most common chronic liver disorder [68]. Therefore, NAFLD has been referred to as the metabolic syndrome manifestation in the liver, with IR being an important trigger for the development of these conditions [69,70]. 

Inflammasome component dysregulation can be related to both obesity [71,72] and NASH [23,73], which in turn may be associated with liver cancer progression. Released DAMPs and PAMPs from these metabolic disorders, such as uric acid, cholesterol, and LDL (low-density lipoprotein), can activate RRPs and initiate an inflammatory response from the trigger of the assembly of inflammasomes, resulting in the production of IL-1β and IL-18 [71]. This low-grade chronic inflammation contributes to NASH and HCC progression. It was already reported that, in response to AT inflammation, adipocytes upregulate IL-1β expression and that IL-1β may mediate IR in liver-derived cells [74]. Nov and colleagues showed that high-fat-fed (HFF) mice had an increase in IL-1β specifically in portal veins compared to systemic blood [75]. They also demonstrated that the absence of IL-1β in HFF mice exhibited no increase in the adipose expression of the pro-inflammatory genes (including macrophage M1 markers), while in wild-type HFF mice the expression of these genes was increased [75]. IL-1β supports ectopic fat accumulation in hepatocytes and AT macrophages, contributing to impaired fat–liver crosstalk in nutritional obesity [75]. IL-18 has also been associated with obesity, and it may contribute to the liver disease development associated with IR [76].

Free fatty acids (FFAs) secreted by hypertrophic adipocytes influence the polarization status of the macrophages. The activation of these immune cells interferes in the progression of metabolic dysfunction in obese individuals. AT macrophages respond to obesity and dietary stimuli by inducing inflammatory processes that are associated with disease progression locally and in the liver. In addition, Kupffer cells are known to play roles in lipid metabolism and macrophage activation [77]. Furthermore, other immune cells are influenced by the obese phenotype. Bijnen and colleagues showed that visceral adipose tissue obtained from obese mice displayed enhanced expression of neutrophil chemotaxis genes. In addition, they presented that neutrophil marker genes were elevated in the hepatic tissue derived from these animals. This elegant study pictured the neutrophils as possible players in the progression of the hepatic pathologies associated with obesity [78]. In a murine model-based study, Grohmann and others showed that obesity induces hepatic STAT-1 signaling and that this signal transduction is key to T cell infiltration within hepatic tissue [79]. It has also been shown that aggregates of these lymphoid cells impact the progression of NASH by the successive induction of hepatic inflammation, triggering a tissue damage and repair cycle [66]. All these immune cell recruitment processes contribute to a chronic inflammatory state and liver disorder. 

## 5. Obesity-Associated NASH IL-1β and IL-18 in Liver Cancer Progression

NASH is a damaging condition associated with advanced liver disease [80]; it is associated with necroinflammation and hepatocyte injury in the steatotic liver [68]. This hepatic pathology displays histological characteristics similar to alcoholic hepatitis, with exacerbated fat deposition and storage in the liver parenchymal cells, features that promote inflammation and necrosis in the liver tissue [81]. These features place NASH as a predisposing condition for developing end-stage liver disease and cardiovascular disorders [67]. NASH patients are known to have increased saturated fat intake and low polyunsaturated fat consumption [68]. 

Dysbiotic gut signals and deranged visceral AT impact tissue and system inflammatory status [82] trigger disruptions that lead to organ fibrosis. Liver fibrosis is generated by the deposition of an extracellular matrix by hepatic stellate cells (HSCs), hepatocytes, and immune cells [83,84], influenced by alterations in miRNAs, systemic cytokines and adipokines, and microenvironmental interactions in the liver [83,85,86,87,88,89]. As fibrotic lesions progress within a hepatocellular damage scenario, the regenerating cells become trapped in dense bands of scar tissue, leading to the formation of hepatic nodules [90]. The common consequences of liver fibrosis occurrence are cirrhosis and HCC. 

Cytokines, chemokines, and eicosanoids support persistent immune cell infiltration and hepatocellular damage. Chronic inflammatory responses in the hepatic tissue cope with liver structure and function deterioration as this induces hepatocyte cell death [91] and disrupts proper tissue repair. Research has shown that active NLRP3 inflammasome induces inflammation and NASH development [92]. Csak and colleagues demonstrated that palmitic acid stimuli induced apoptosis and danger signals in hepatocytes, which thus stimulate the Kupffer cells’ inflammasome [93]. In addition, increasing data indicate that the NLRP3 inflammasome is stimulated by lipotoxicity and contributes to NASH development in mice [22]. Augmented levels of the inflammasome-derived IL-1β are very relevant for the worsening of the inflammatory status during NASH onset, as shown by murine model experiments [94]. IL-1 activates HSCs, resulting in the progression from liver injury to fibrogenesis [95]. In addition, in a hypoxic microenvironment, IL-1β modulates via macrophages and the IL-1β/HIF-1α/COX2 axis, supporting the transition of the epithelial-mesenchymal transition in HCC cells [96]. 

Studies with IL-18 are controversial. Yamanishi and colleagues showed that IL-18 knockout mice developed dyslipidemia resulting in NASH [97]. The intravenous administration of IL-18 significantly improved dyslipidemia, inhibited body weight gain, and prevented the onset of NASH in these mice [97]. On the other hand, a recent study demonstrated that IL-18 receptor knockout mice were protected from early liver damage and that IL-18 signaling is pivotal for the initiation of liver injury in murine NASH. In humans, IL-18 was elevated in the serum of NASH patients [98] and in obese children with advanced liver steatosis [99]. However, another study showed an absence of alterations in the circulating IL-18 levels of male subjects with NAFLD [100]. 

Another important participant in the liver disease progression to NASH that may be involved in liver cancer is the microbiome composition [101,102]. The gut microbiota is a diverse microbial community that dwells in the gastrointestinal tract (GIT). It is composed of bacteria, archaea, viruses, and eukaryotic microbes and can influence mucosal and system immunity through the secretion of immunomodulatory components [103]. The imbalance in the variety of species and metabolites secreted, called dysbiosis, disturbs gastrointestinal homeostasis. The intestine and liver are organs placed close to each other; many gut-associated molecules and gut bacteria factors impact strongly on hepatic disease. Alterations of gut microbiome homeostasis are known to occur in obese individuals, whose diet directly influences the intestinal microbiota by changing the microbiome gene expression profile and altering the relative abundance of specific microbes [104]. Intestinal dysbiosis is expected to influence NASH progression in affected individuals once patients with NASH display enhanced intestinal permeability [105], aberrant overgrowth of intestinal bacteria [106], and higher endotoxin LPS levels in portal blood [107]. NLRP6 inflammasome is found in intestinal epithelial cells and plays an important role in intestinal homeostasis and mucus secretion regulation in the intestinal goblet [23]. Dysregulation in NLRP3 and NLRP6 inflammasomes, and consequently in IL-1 β and IL-18, can lead to NASH, which enables gut dysbiosis occurrence and portal circulation homeostasis disruption [16].

**Figure 2:**
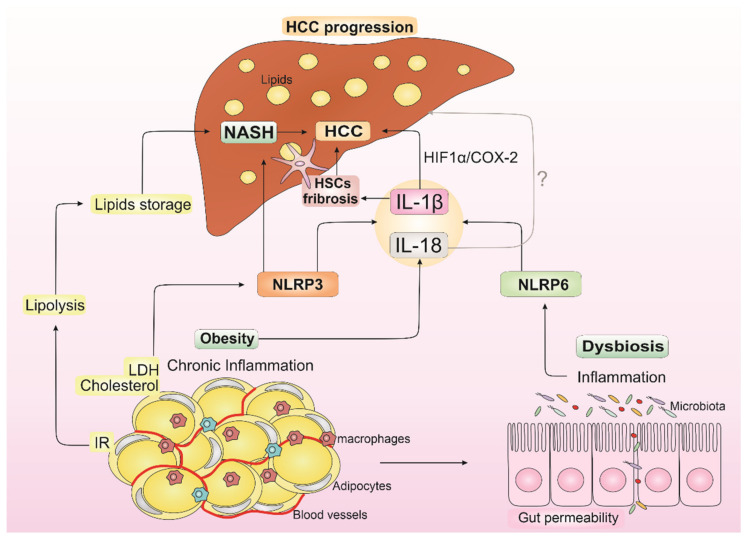
**Impact of IL-1β and IL-18 from obesity, dysbiosis, and non-alcoholic steatohepatitis (NASH) in hepatocellular carcinoma (HCC) progression.** Insulin resistance (IR) from obesity increases lipolysis, which leads to liver lipid storage and contributes to NASH establishment. LDL (low-density lipoprotein) and cholesterol from obesity can activate NLRP3 inflammasome, contributing to NASH development and resulting in IL-1β release. IL-18 is also released in the obesity state. Dysbiosis is known to occur in obese individuals with dysregulation in NLRP6 inflammasome and consequent IL-1β and IL-18 releasing. IL-1β can modulate hepatic stellate cells (HSCs), resulting in the progression from liver injury to fibrogenesis, which contributes to HCC development and supports epithelial-mesenchymal transition in HCC cells through IL-1β/HIF-1α/COX2 axis in hypoxic conditions. In turn, the role of IL-18 in NASH, and consequently in HCC progression, is controversial in the literature (the symbol “?” indicates the uncertain role of IL-18 in HCC progression). This inflammatory environment supported by IL-1β and IL-18 from obesity, dysbiosis, and NASH promotes an increase in cells with genetic alterations, creating a pro-tumorigenic microenvironment that may induce HCC development.

All these factors contribute to the occurrence of chronic liver inflammation and injurious stimuli driven by mediators secreted by liver-residing immune cells, such as macrophages and liver-specific Kupffer cells (KCs) [108]. Furthermore, this inflammatory environment supported by obesity, dysbiosis, and NASH promotes an increase in cells with genetic alterations, creating a pro-tumorigenic microenvironment that supports HCC development (Figure 2) [109].

## 6. Modulating IL-1β and IL-18 as a Therapeutic Target in HCC

Treatment for HCC depends on the disease’s stage. In the early phases, surgery, ablation, or radiofrequency is generally recommended [110]. In the intermediate stage, transarterial chemoembolization (TACE) [111] or locoregional or systemic therapy [112] is suggested. Systemic therapies are also applied for managing advanced disease stages, such as tyrosine kinase inhibitors (ex., sorafenib), ICIs, such as atezolizumab (anti-PDL1), and other monoclonal antibodies, including bevacizumab, which blocks the endothelial growth factor (anti-VEGF) [1]. The combination of two or more drugs can increase the therapy effectiveness. Despite this variety, the treatments rarely completely remove the tumor, especially in later stages of the malignancy [113].

HCC initiation and progression is deeply linked with changes in the hepatic microenvironment, with cytokines playing key roles [114]. Due to high resistance to chemotherapy and the loss of response to the current immunotherapy presented by this cancer, there is a multitude of new immunotherapies in development with varying degrees of success. One particularly important consideration when administering this kind of therapy is the adequate patient selection, as this modality of treatment is often very expensive and has severe potential hazards for the subjects. 

Cancer treatments have been interposed by numerous practical issues, such as low intratumoral concentration, high toxicity, and immune checkpoint induction [115]. Another difficulty, specifically with regard to HCC, is its complex microenvironment—which changes not only with disease progression but also with disease background [114], and such differences dictate the success of the therapy [116]. Concerning cytokine therapy in HCC, the only one clinically approved with somewhat frequent use is interferon (IFN) for HCC in patients with a hepatitis background—which is not a standard of care even with this clinical background [116].

Promising cytokine immunotherapies currently under investigation are pegylated INF [117], INF-β [118], IL-2 in adenoviral vector [119], IL-6 [120], IL-7 [121], TNF-α [122], and TGF-beta [123]. In addition, intracellular biosignaling pathways related to cytokines are also a promising approach [124].

Pre-clinical evidence suggests that IL-1β induces PD-1L expression in malignant cells [51], which is an immunological escape strategy, and promotes metastization, as well as high levels of serum IL-1β, which correlates with poor patient prognosis. The inhibition of NLRP3 inflammasome both reduces IL-1β and malignant cell growth, migration, and invasion and induces apoptosis [125]. Thereby, inhibiting IL-1β may be investigated as a potential HCC therapy. On the other hand, inhibiting IL-18 seems to be dual. A study showed that the inhibition of IL-18 through TLR2 alleviated mouse HCC progression, whereas deletion of the IL-18 receptor enhanced tumor growth once the IL-18 had tumor-suppressive effects by promoting tumor-infiltration T cells [57].

**Figure 3:**
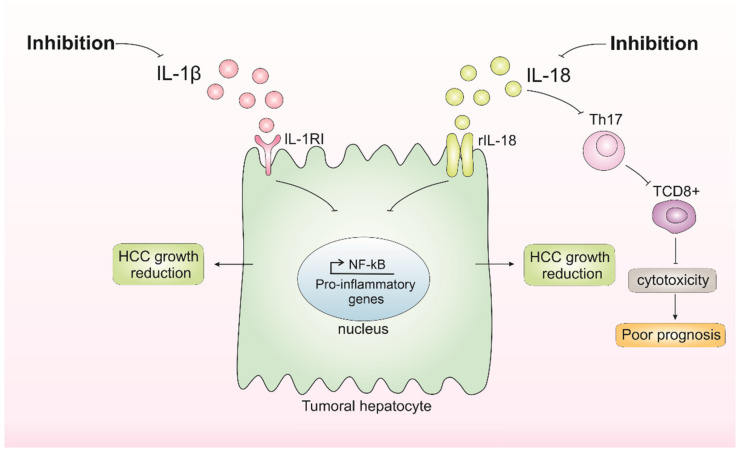
**Inhibition of IL-1β and IL-18 and its consequences in hepatocellular carcinoma (HCC) growth.** Inhibition of IL-1β results in the inhibition of pathways affected by this cytokine, such as nuclear factor kappa B (NF-κB), which possibly results in the inhibition of tumor growth. Inhibition of IL-18 appears to have dual consequences, either reducing HCC growth or favoring poor prognosis by impairing tumor-infiltration T CD8^+^ cells.

According to these studies, modulating IL-1β and IL-18 seems promising (Figure 3). Overall, the future of HCC treatment modulating IL-1β and IL-18 should take into account the different backgrounds and a deep understanding of tumor biology and its interplay with the microenvironment integrated in a personalized medicine approach. For this, additional studies exploring different models and backgrounds are necessary. Moreover, further investigating the impact of IL-18 in the HCC context is needed to better understand the role of inflammasome-mediated cytokines in HCC initiation and progression. 

## 7. Concluding Remarks

HCC is an aggressive cancer that can be triggered by many different risk factors, including NASH and obesity. The understanding of the connection of these disorders to liver cancer remains poorly understood. However, once inflammasome dysregulation is associated with NASH, obesity, and HCC, the cytokines released by them could be a possible link between obesity-associated NASH and HCC development. IL-1β is capable of influencing many consequences in obesity-associated NASH patients that can lead to HCC development, as shown herein. However, IL-18 can also modulate the liver microenvironment, displaying a dual role in HCC. Considering the importance of IL-1β and IL-18 in obesity-associated NASH and, consequently, in HCC progression, their therapeutic exploration appears to be promising for the management of this lethal cancer. 

## Data Availability

Not applicable.
